# Nicotine induces endothelial dysfunction and promotes atherosclerosis via GTPCH1

**DOI:** 10.1111/jcmm.13812

**Published:** 2018-08-09

**Authors:** Jingyuan Li, Shangming Liu, Guangqing Cao, Yuanyuan Sun, Weiqian Chen, Fajin Dong, Jinfeng Xu, Cheng Zhang, Wencheng Zhang

**Affiliations:** ^1^ The Key Laboratory of Cardiovascular Remodeling and Function Research, Chinese Ministry of Education and Chinese Ministry of Health; The State and Shandong Province Joint Key Laboratory of Translational Cardiovascular Medicine Qilu Hospital of Shandong University Jinan Shandong China; ^2^ Department of Histology and Embryology Shandong University School of Medicine Jinan China; ^3^ Department of Cardiovascular Surgery Qilu Hospital of Shandong University Jinan Shandong China; ^4^ Departmen of Cardiovascular Surgery of the First Affiliated Hospital & Institute for Cardiovascular Science Soochow University Suzhou China; ^5^ Department of Ultrasonography Second Clinical College of Jinan University Shenzhen People's Hospital Shenzhen China

**Keywords:** atherosclerosis, GTPCH1, HuR, nicotine

## Abstract

Smoking is a major preventable risk factor for atherosclerosis. However, the causative link between cigarette smoke and atherosclerosis remains to be established. The objective of this study is to characterize the role of GTP cyclohydrolase 1 (GTPCH1), the rate‐limiting enzyme for de novo tetrahydrobiopterin (BH4) synthesis, in the smoking‐accelerated atherosclerosis and the mechanism involved. In vitro, human umbilical vein endothelial cells were treated with nicotine, a major component of cigarette smoke, which reduced the mRNA and protein levels of GTPCH1 and led to endothelial dysfunction. GTPCH1 overexpression or sepiapterin could attenuate nicotine‐reduced nitric oxide and ‐increased reactive oxygen species levels. Mechanistically, human antigen R (HuR) bound with the adenylateuridylate‐rich elements of the GTPCH1 3′ untranslated region and increased its stability; nicotine inhibited HuR translocation from the nucleus to cytosol, which downregulated GTPCH1. In vivo, nicotine induced endothelial dysfunction and promoted atherosclerosis in ApoE^−/−^ mice, which were attenuated by GTPCH1 overexpression or BH4 supplement. Our findings may provide a novel and promising approach to atherosclerosis treatment.

## 1 INTRODUCTION

Cigarette smoking has been implicated in numerous diseases and accounts for 175 000 cardiovascular deaths annually in the United States.^1^ Especially, smoking is a major preventable risk factor for atherosclerosis, the leading cause of death among the cardiovascular diseases. Nicotine is a predominant chemical in cigarette smoke and has been suspected to be a causative agent for atherosclerosis for decades.^2^ Although some work has explored the link between smoking and atherosclerosis, the mechanism of the pro‐atherogenic action of nicotine is still largely speculative.

Endothelial dysfunction is an early key event during the development of atherogenesis^3^ and is characterized by the reduced bioavailability of nitric oxide (NO). In endothelial cells, NO is a free radical gas generated from the metabolism of L‐arginine by endothelial NO synthase (eNOS).^4^ A critical determinant of eNOS activity is the availability of tetrahydrobiopterin (BH4). BH4 deficiency uncouples eNOS to generate superoxide rather than NO.^5^ GTP cyclohydrolase 1 (GTPCH1) is the rate‐limiting enzyme in the de novo biosynthetic pathway of BH4. GTPCH1 inhibition leads to a rapid decrease in BH4 level and consequent eNOS uncoupling.^6^ GTPCH1 deficiency is an important mechanism of endothelial dysfunction in cardiovascular diseases and diabetes.^7^, ^8^ Considering the vital role of GTPCH1, investigation is needed of the mechanism involved in regulating GTPCH1 gene expression.

GTPCH1 is regulated by several mechanisms, including transcription and post‐translational modifications,^9^ and association with the GTPCH feedback regulatory protein (GFRP), which inhibits GTPCH1 activity.^10^ Human antigen R (HuR) is an RNA‐binding protein that regulates the stability of adenylate‐uridylate‐rich element (ARE)‐containing mRNAs such as plasminogen activator inhibitor 2 and vascular endothelial growth factor to enhance translation.^11^, ^12^, ^13^ This is an important pathway for HuR to regulate the expression of target genes. However, whether GTPCH1 mRNA is regulated by HuR is unknown.

In this study, we investigated whether GTPCH1 is a target of HuR and whether downregulation of GTPCH1 in endothelial cells mediates the endothelial dysfunction induced by nicotine. Nicotine promoted atherosclerosis in ApoE^−/−^ mice, which was attenuated by GTPCH1 overexpression or BH4 supplementation.

## 2 METHODS AND MATERIALS

### 2.1 Materials

Nicotine, sepiapterin, BH4, actinomycin D (ActD), acetylcholine (ACh), and phenylephrine (PE) were from Sigma‐Aldrich (St. Louis, MO). CMLD‐2 was from Calbiochemical (San Diego, CA). The Magna RIP kit was from Millipore (Billerica, MA). Nucleoprotein Extraction Kit was from Sangon Biotech (Shanghai, China). Commercial kits for determining NO and malondialdehyde (MDA) were from Jiancheng Bioengineering Institute (Nanjing, China). Lentivius expressing GFP or GTPCH1 (LV.GFP and LV.GTPCH1) was generated by Vigenebio Co. (Jinan, China).

### 2.2 Cell culture

Human umbilical vein endothelial cells (HUVECs) were obtained from Clonetics (Walkersville, MD). Cells were grown in endothelial basal medium supplemented with 2% foetal bovine serum, 100 U/mL penicillin and 10 mg/mL streptomycin. Cultured cells between passages three and eight were used for experiments. All cells were incubated in a humidified atmosphere of 5% CO_2_ and 95% air at 37°C. When 70%‐80% confluent, cells were treated with nicotine at the indicated dose.

### 2.3 Western blot analysis

Protein extracts were prepared by lysing cells in RIPA lysis buffer (Santa Cruz Biotechnology). Cleared lysates were separated on 10% Tris‐glycine gels, then transferred to nitrocellulose membranes, which were blocked in 5% skim milk, then incubated with primary antibodies for GAPDH (1:1000, Cell Signaling Technology, USA), GTPCH1 (1:1000, Sigma); HuR (1:1000) and p‐HuR S221 (1:1000, Millipore); and Lamin B1 (1:1000, Cell Signaling Technology). After a wash and incubation with horseradish peroxidase‐conjugated secondary antibodies, protein bands were visualized using chemiluminescent HRP substrate (Millipore, USA). The intensity of individual bands was measured by densitometry (Model GS‐700, Imaging Densitometer; Bio‐Rad, Hercules, CA). All experiments were repeated four times and mean values were derived.

### 2.4 Quantitative real‐time PCR (RT‐PCR)

Total RNA was extracted from cells (kept at −80°C) using RNA Extraction Reagent (Vazyme Biotech Co., Nanjing), and 1 μg of RNA was reverse‐transcribed into cDNA using Hiscript Reverse Transcriptase (Vazyme Biotech Co.). PCR amplification involved the SYBR PCR mix (Bio‐Rad). The oligonucleotide primer sequences were for GTPCH1, 5′‐CCAGGTGCAGCAATGGGTTC‐3′ and 5′‐TTCAACCACTACCCCGACTC‐3′; and GAPDH, 5′‐AGCTAAGAGAAGGGCGGAAC‐3′ and 5′‐CATCTGCAGGCTGACATTGA‐3′.

### 2.5 Detection of reactive oxygen species (ROS) and intracellular BH4

Intracellular ROS levels were measured by dihydroethidium (DHE) fluorescence. Briefly, cells were incubated with 10 μmol/L DHE for 30 minutes, then washed with serum‐free DMEM for three times. Dihydroethidium fluorescence intensity was recorded using a fluorescent reader at 480 nm excitation and 525 nm emission. Concentrations of BH4 were measured using an ELISA kit.

### 2.6 RNA immunoprecipitation (IP) assays

The Magna RIP kit was used for RNA IP assay. Briefly, whole‐cell lysates were incubated at 4°C overnight with magnetic protein A/G beads precoated with 5 μg rabbit IgG or HuR antibody (Millipore). Beads were washed and incubated with proteinase K buffer (30 minutes at 55°C), followed by RNA isolation from the immunoprecipitates, then cDNA synthesis. PCR involved use of the primers for quantitative RT‐PCR.

### 2.7 Animals and experimental protocols

Male ApoE^−/−^ mice (C57BL/6J genetic background, 8‐12 weeks old) were from Vital River Laboratories (Distributor of Jackson Laboratory, Beijing) and housed under specific pathogen‐free conditions on a 12‐hour light/12‐hour dark cycle with food and water freely available. The procedures were in accordance with institutional guidelines and were approved by the Animal Care and Use Committee of Shandong University.

In the first part of the animal study, ApoE^−/−^ mice fed a high‐fat diet (HFD; 47% calories from fat and 0.2% from cholesterol) were divided randomly into four groups (n = 10 in each group): (a) the sham group fed an HFD, (b) the nicotine group fed an HFD+nicotine (100 mg/L in drinking water), (c) the BH4 group fed an HFD and received BH4 (10 mg/kg per day, intraperitoneal injection) and (d) the nicotine+BH4 group fed an HFD+nicotine and received BH4.

In the second part of the animal study, ApoE^−/−^ mice fed an HFD were divided randomly into four groups (n = 10 in each group) and received the following treatments for 12 weeks: (a) the GFP group injected intravenously with LV.GFP (1 × 10^9^ plaque‐forming units [PFU] in 200 μL phosphate buffered saline) and fed an HFD, (b) the GFP+nicotine group injected intravenously with LV.GFP and fed an HFD+nicotine, (c) the GTPCH1 group injected intravenously with LV.GTPCH1 (1 × 10^9^ PFU) and fed an HFD and (d) the GTPCH1+nicotine group injected intravenously with LV.GTPCH1 and fed an HFD+nicotine.

### 2.8 Measurement of endothelium‐dependent vasorelaxation

The endothelium‐dependent vasorelaxation was measured as follows. Mice were anaesthetized with isoflurane (2%) and isolated aorta rings were immersed in Krebs bicarbonate buffer (118 mmol/L NaCl, 4.7 mmol/L KCl, 25 mmol/L NaHCO_3_, 1.2 mmol/L KH_2_PO_4_, 1.2 mmol/L MgSO_4_, 2.5 mmol/L CaCl_2_, and 5 mmol/L glucose) and then suspended by two tungsten wires mounted in a vessel myograph system (Danish Myotechnologies, Aarhus, Denmark). After undergoing an equilibration period, rings were treated with 1 μmol/L phenylephrine for induction of contraction. At the plateau of contraction, acetylcholine (ACh) (10^−8^‐10^−4^ mol/L) was added to elicit endothelium‐dependent vasorelaxation. The relaxation was calculated as a ratio of ACh‐induced vasodilation to phenylephrine‐elicited vasoconstriction, and the ratio at one was set to 100% of relaxation.

### 2.9 Oil‐red O staining

Atherosclerotic lesions were quantified in aortic‐root cross sections from frozen OCT‐embedded hearts. Briefly, serial 6‐μm‐thick cryostat sections were prepared from the origin of the aortic valve cusps, and cross‐sectional analysis for atherosclerotic lesion was performed every 60 μm over 360 μm by staining with Oil‐red O (Sigma‐Aldrich). The extent of atherosclerosis was expressed as the percentage of aortic root covered by lesions. Images were captured digitally with a video camera and analysed using Image‐Pro Plus (Media Cybernetics). Samples were coded, and acquisition of images and analysis of lesions were performed in a blinded fashion.

### 2.10 Histological analysis of aortic lesions

The thoracic aorta was fixed in 4% paraformaldehyde overnight, embedded in paraffin and sectioned at 4 μm. The deparaffinized, rehydrated sections from thoracic aortas and cryosections from aortic roots (5 μm) were microwaved in citrate buffer for antigen retrieval, incubated in endogenous peroxidase (DAKO) and protein block buffer and then primary antibodies overnight at 4°C. Slides were rinsed with washing buffer and incubated with labelled polymer‐horseradish peroxidase‐conjugated anti‐mouse/anti‐rabbit antibodies followed by DAB+ chromogen detection (DAKO). Data were analysed using Image‐Pro Plus.

### 2.11 Measurements of serum NO and MDA

Serum NO and MDA levels were assayed using commercial kits.

### 2.12 Statistical analysis

All analyses involved use of SPSS v23 (SPSS Inc., Chicago, IL). Data are represented as mean ± SD. Comparison of two groups of continuous data involved Student *t* test and multiple groups one‐way ANOVA, followed by the Scheffe post‐hoc test. Statistical significance was determined at *P* < 0.05.

## 3 RESULTS

### 3.1 Nicotine inhibits GTPCH1 expression in endothelial cells

**Figure 1:**
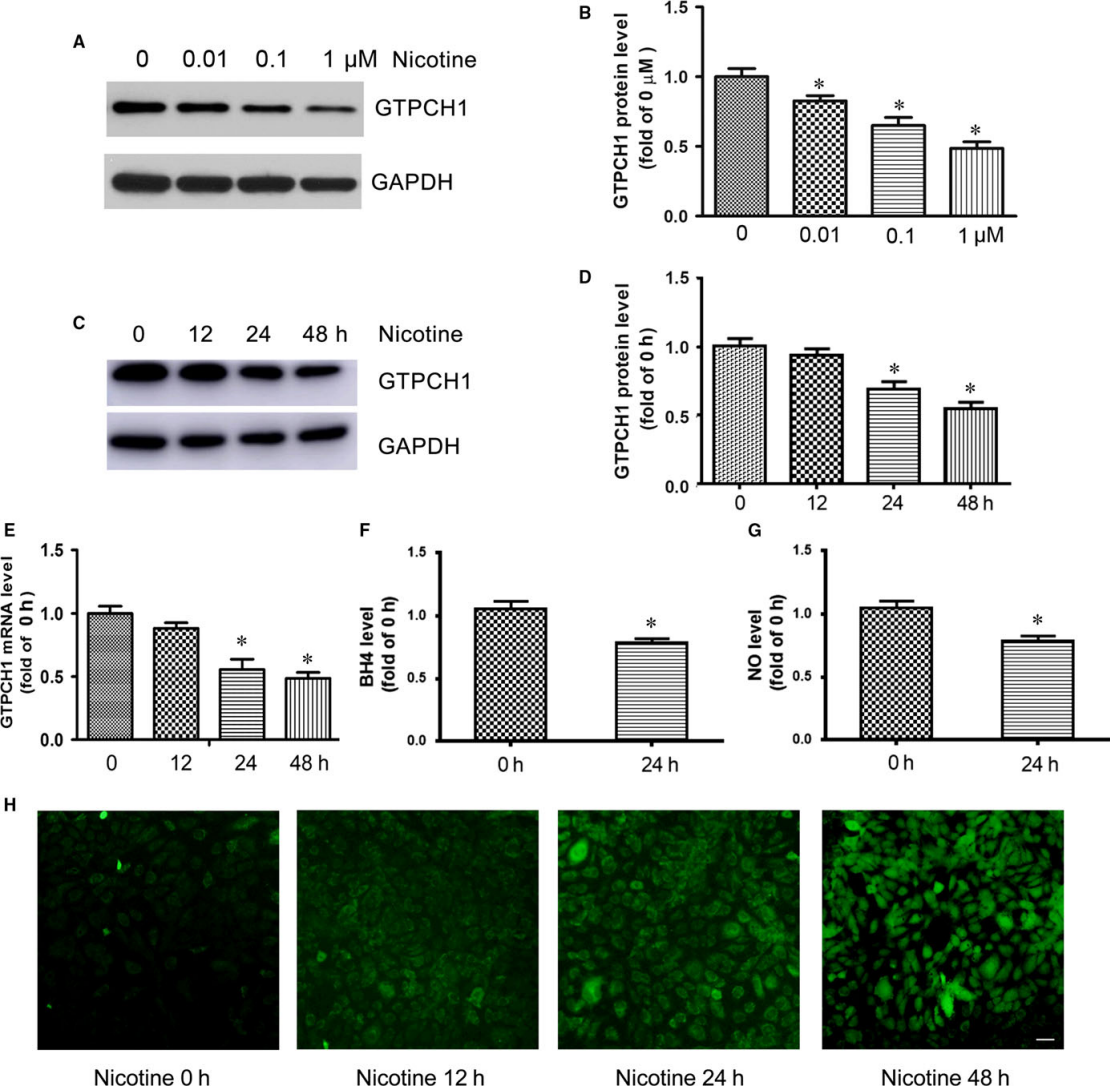
Nicotine inhibits GTPCH1 expression in endothelial cells. A, Western blot analysis of GTPCH1 expression in HUVECs treated with different doses of nicotine for 48 h and B, quantitative analysis (n = 4). *P < 0.05 vs 0 μmol/L. C, Western blot analysis of GTPCH1 expression in HUVECs treated with 1 μmol/L nicotine for different times and D, quantitative analysis (n = 4). *P < 0.05 vs 0 h. E, Quantitative RT‐PCR analysis of GTPCH1 mRNA expression in HUVECs treated with 1 μmol/L nicotine for different times (n = 4). *P < 0.05 vs 0 h. F, BH4 level in HUVECs treated with nicotine for 24 h (n = 3). *P < 0.05 vs 0 h. G, NO level in HUVECs treated with nicotine for 24 h (n = 3). *P < 0.05 vs 0 h. H, Detection of reactive oxygen species (ROS) levels by green fluorescence in HUVECs treated with nicotine for different times. Scale bar = 50 μm. Data are mean ± SD

GTPCH1 is important for BH4 synthesis, which contributes to NO production and endothelial function. To investigate the effect of nicotine on GTPCH1 expression, HUVECs were treated with different doses of nicotine for 48 hour. GTPCH1 protein level was decreased by nicotine in dose‐dependent manner (Figure 1A and B). Besides, HUVECs were exposed to 1 μmol/L nicotine for different times. GTPCH1 protein level was significantly decreased by nicotine during 48 hour of exposure (Figure 1C), with maximal 2‐fold reduction at 48 hour (Figure 1D). Nicotine also decreased GTPCH1 mRNA level (Figure 1E). As expected, the levels of BH4 in endothelial cells and NO in supernatant were reduced after nicotine exposure (Figure 1F and G).

Considering about blood vessels in the body exposure 5%‐7% O_2_ based on vessel type, HUVECs were cultured in the 5% O_2_ incubator and treated with 1 μmol/L nicotine. The levels of GTPCH1, BH4 and NO were also decreased by nicotine in the environment with low oxygen (Figure S1).

Under conditions of limited BH4, eNOS functions in an “uncoupled” state in which reduced nicotinamide‐adenine dinucleotide phosphate‐derived electrons, rather than L‐arginine, are added to molecular oxygen, which leads to ROS production.^5^ Therefore, we evaluated the effect of nicotine on ROS production. As expected, nicotine time‐dependently increased the levels of ROS in HUVECs (Figure 1H). Taken together, nicotine inhibited GTPCH1 expression and increased ROS production in endothelial cells.

### 3.2 GTPCH1 overexpression rescues nicotine‐reduced BH4 and ‐increased ROS levels

**Figure 2:**
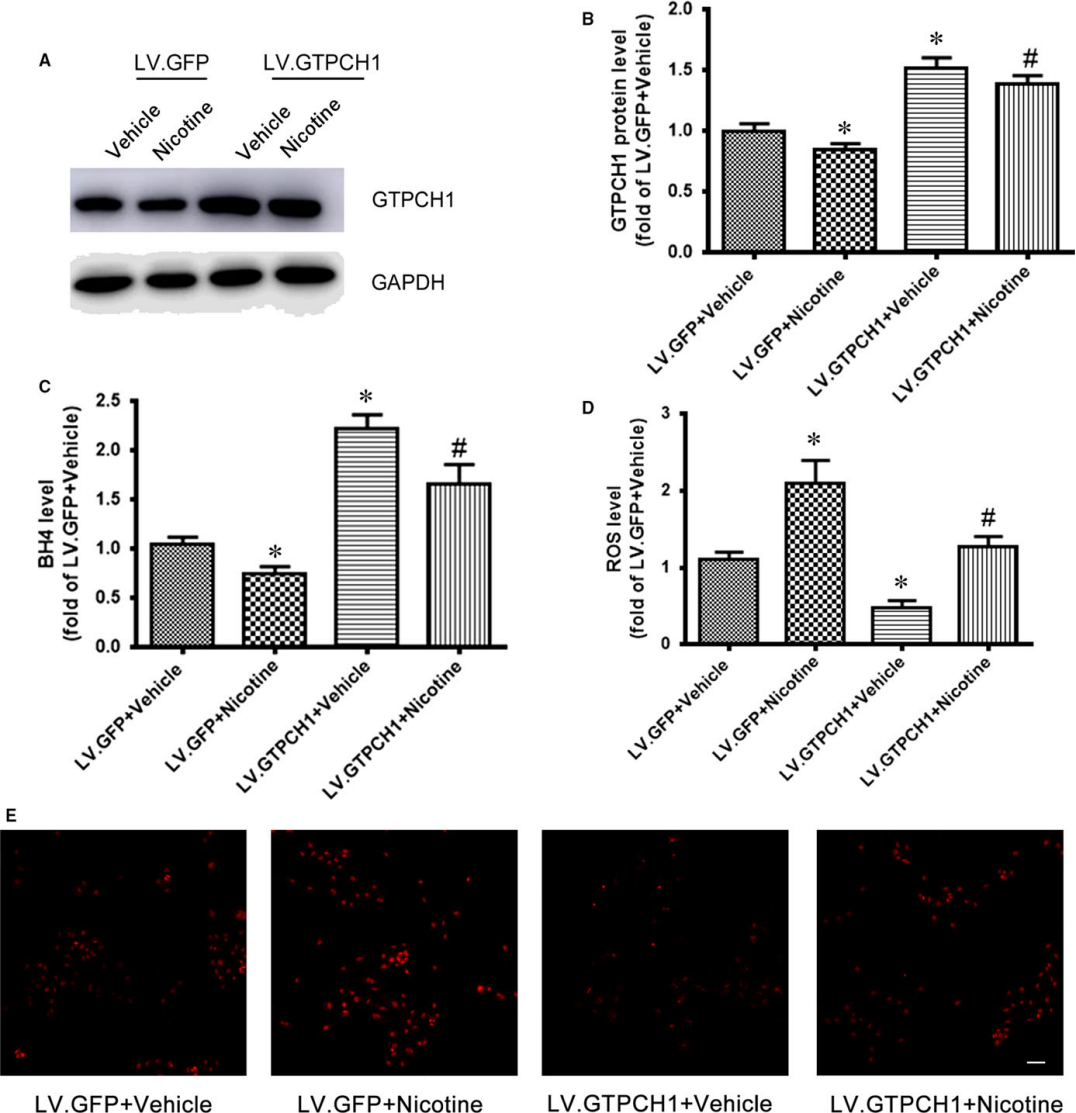
GTPCH1 overexpression rescues nicotine‐reduced BH4 and ‐increased reactive oxygen species (ROS) levels. A, HUVECs were transfected with lentivirus harbouring GFP or GTPCH1 for 48 h, then treated with 1 μmol/L nicotine for 24 h. Western blot analysis of GTPCH1 and B, quantitative analysis (n = 4). *P < 0.05 vs LV.GFP+Vehicle. ^#^ *P* < 0.05 vs LV.GFP+Nicotine. C and D, Levels of BH4 (C) and NO (D) in HUVECs (n = 4). *P < 0.05 vs LV.GFP+Vehicle. ^#^ *P* < 0.05 vs LV.GFP+Nicotine. E, Detection of ROS levels by red fluorescence in HUVECs. Scale bar = 100 μm. Data are mean ± SD

To determine the role of GTPCH1 in the nicotine‐reduced BH4 level, HUVECs were infected with a lentivirus‐expressing GFP (LV.GFP) or LV.GTPCH1 for overexpression, then treated with nicotine. LV.GTPCH1 increased the GTPCH1 level in HUVECs with or without nicotine treatment (Figure 2A and B). Meanwhile, GTPCH1 overexpression rescued the nicotine‐reduced BH4 level in HUVECs (Figure 2C). Also, nicotine‐increased ROS levels were attenuated by GTPCH1 overexpression (Figure 2D and E). These results confirm the role of GTPCH1 in nicotine‐reduced BH4 and ‐increased ROS levels. Thus, nicotine exposure inhibited GTPCH1 levels, which reduced BH4 and increased ROS levels.

### 3.3 Sepiapterin attenuates nicotine‐reduced NO and ‐increased ROS levels

**Figure 3:**
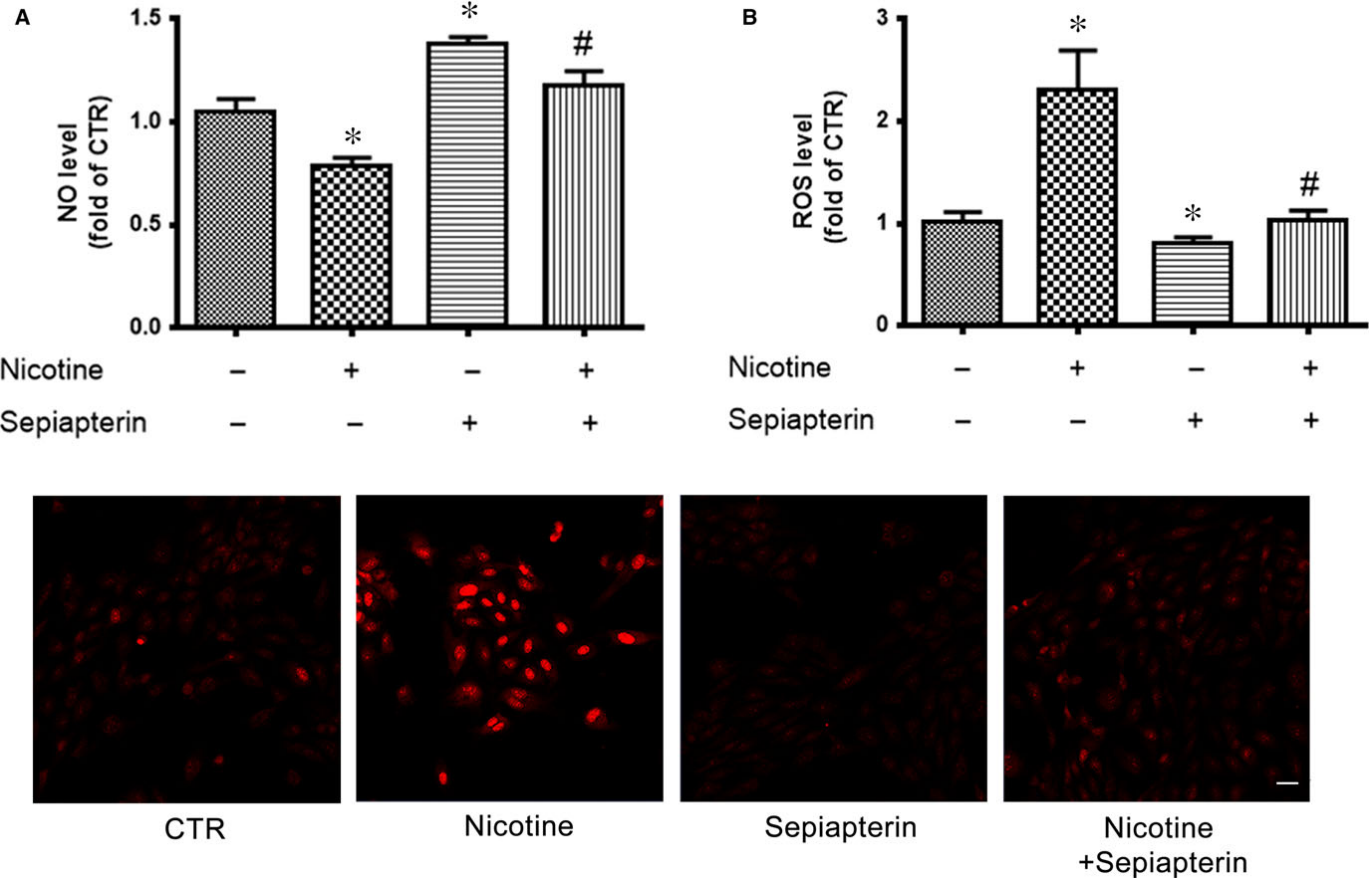
Sepiapterin attenuates nicotine‐reduced NO and ‐increased reactive oxygen species (ROS) levels. A, NO levels were measured in HUVECs pretreated with 10 μmol/L sepiapterin for 30 min, then nicotine for 24 h. (n = 4). *P < 0.05 vs control (CTR). ^#^ *P* < 0.05 vs Nicotine. B and C, ROS levels measured by DHE fluorescence intensity in HUVECs (C) and quantitative analysis (B). n = 4, *P < 0.05 vs CTR. ^#^ *P* < 0.05 vs Nicotine. Scale bar = 50 μm. Data are mean ± SD

Because BH4 is a cofactor for eNOS and contributes to NO production, we detected whether BH4 supplement could rescue the nicotine‐reduced NO level. Human umbilical vein endothelial cells were pretreated with sepiapterin, a stable precursor of BH4, then exposed to nicotine. Nicotine decreased NO production, which was rescued by sepiapterin pretreatment (Figure 3A). Similarly, nicotine‐increased ROS levels were attenuated by sepiapterin (Figure 3B and C). These results further support the roles of BH4 in nicotine‐reduced NO and ‐increased ROS levels.

### 3.4 Nicotine regulates GTPCH1 by suppressing HuR translocation

**Figure 4:**
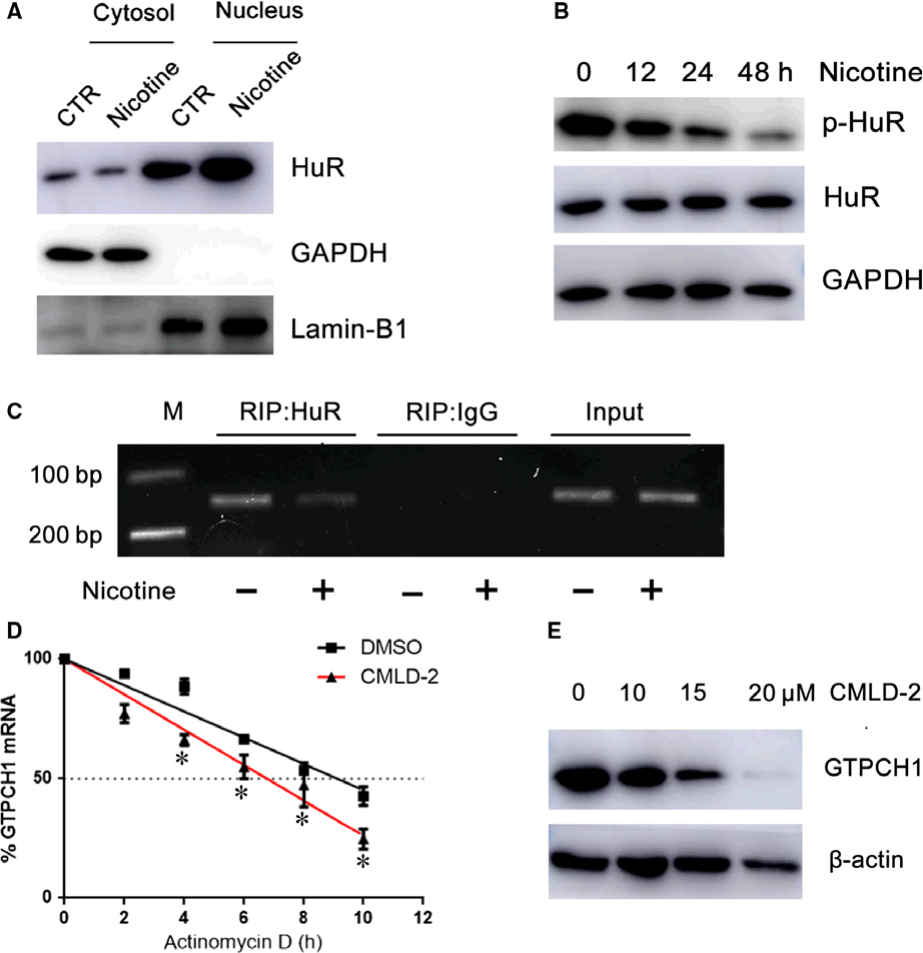
Nicotine regulates GTPCH1 by suppressing HuR translocation. A, Western blot analysis of HuR distribution and B, phospho‐HuR (p‐HuR) S221 and total HuR levels in HUVECs treated with nicotine. C, RNA immunoprecipitation with anti‐HuR antibody or control IgG. D, HUVECs were treated with DMSO or CMLD‐2 (10 μmol/L) for 1 h, then 10 μg/mL actinomycin D. Proportion of GTPCH1 mRNA was determined by RT‐PCR (n = 4). *P < 0.05 vs DMSO. E, Western blot analysis of GTPCH1 in HUVECs treated with CMLD‐2 for 24 h

To better understand the regulation of GTPCH1 expression by nicotine, we examined its 5′ untranslated region (UTR) and 3′ UTR to assess whether GTPCH1 may be a target of posttranscriptional modification. We identified seven conserved adenylate‐uridylate‐rich elements (AREs) in the 3′ UTR of human GTPCH1 mRNA and nine AREs in the 3′ UTR of mouse GTPCH1 mRNA. Thus, GTPCH1 mRNA may be a target of HuR. We first evaluated the effect of nicotine on HuR translocation. Nicotine decreased cytoplasmic HuR level (Figure 4A) but did not alter total HuR level (Figure 4B), so nicotine suppressed HuR translocation from nuclei to cytosol. HuR translocation is regulated by its phosphorylation at S221, which elevates its cytoplasmic abundance.^14^ We detected HuR phosphorylation after nicotine treatment and found that nicotine decreased the level of phospho‐HuR at S221 (Figure 4B), which explained the reduced cytoplasmic HuR level after nicotine treatment.

We assessed the ability of HuR to bind with GTPCH1 mRNA. RNA IP with an anti‐HuR antibody or control IgG showed that HuR could bind to GTPCH1 mRNA and nicotine reduced this binding (Figure 4C). Next, we examined the effect of HuR on the stability of GTPCH1 mRNA. Human umbilical vein endothelial cells were pre‐treated with CMLD‐2, an HuR‐specific inhibitor,^15^ then actinomycin D, a transcriptional inhibitor. The half‐life of GTPCH1 mRNA decreased from 9.5 to 7 hour after HuR inhibition (Figure 4D), so HuR enhanced GTPCH1 mRNA stability. As expected, CMLD‐2 also dose‐dependently decreased GTPCH1 protein level (Figure 4E). These results indicate that nicotine suppressed HuR translation to decrease GTPCH1 level.

### 3.5 BH4 supplement reduces nicotine‐accelerated atherosclerosis

**Figure 5:**
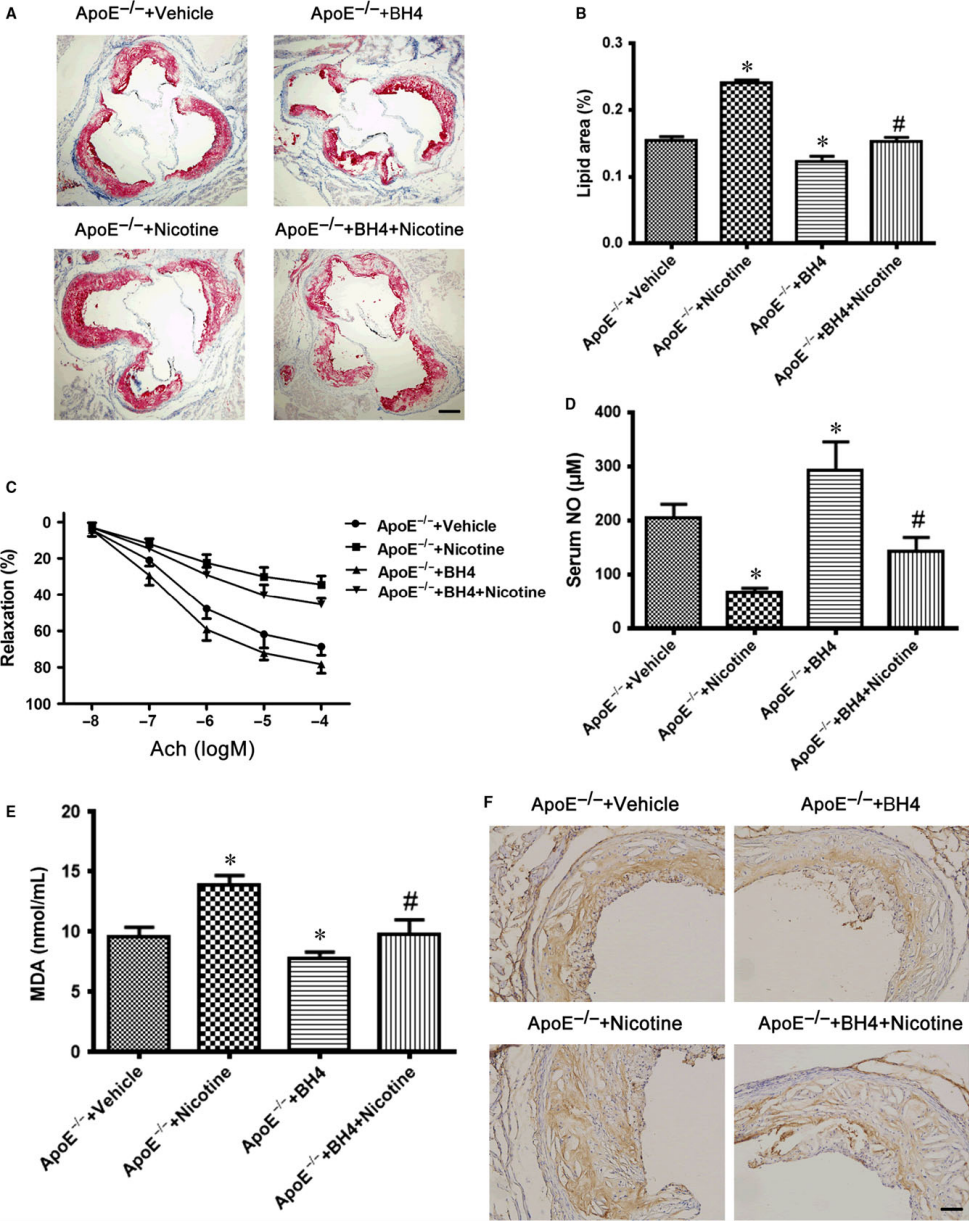
BH4 supplement reduces nicotine‐accelerated atherosclerosis in mice. A, Oil‐red O staining of cross‐sectional aortic root lesions in 4 groups of ApoE^−/−^ mice. Scale bar = 100 μm. B, Quantitative analysis of aortic lesions expressed as percentage lesion area relative to total aortic area. n = 8 per group. *P < 0.05 vs ApoE^−/−^+Vehicle, ^#^ *P* < 0.05 vs ApoE^−/−^+Nicotine. BH4, tetrahydrobiopterin. C, Aortic rings were isolated from the 4 groups of mice. Quantification of vasorelaxation elicited by ACh in an organ bath. n = 5 per group. *P < 0.05 vs ApoE^−/−^+Vehicle. D and E, Serum NO (D) and malondialdehyde (MDA) (E) levels from the 4 groups of mice (n = 6). *P < 0.05 vs ApoE^−/−^+Vehicle, ^#^ *P* < 0.05 vs ApoE^−/−^+Nicotine. F, Representative immunohistochemical staining of 4‐HNE in aortic plaques. Scale bar = 50 μm. Data are mean ± SD

To determine the effect of nicotine and BH4 on the ability to induce atherosclerosis, ApoE^−/−^ mice were fed an HFD supplemented with or without BH4 or nicotine for 12 weeks later, then sacrificed, and the size of atherosclerotic lesions was analysed. The lesion size in aortic roots was greater with nicotine than vehicle treatment (Figure 5A and B), so nicotine accelerated the development of atherosclerosis and was reduced with BH4 treatment (Figure 5A and B). We further determined the effect of BH4 or nicotine on the endothelial function in ApoE^−/−^ mice. ACh‐induced relaxation in the aortic artery was greatly reduced with nicotine treatment and improved with BH4 supplement (Figure 5C). As expected, nicotine also reduced serum NO level and increased serum MDA level in mice (Figure 5D and E). All of these negative nicotine‐induced effects were reversed with BH4 supplement (Figure 5D and E), which further suggests that BH4 is required for nicotine‐mediated uncoupling of eNOS in vivo. Moreover, nicotine increased 4‐hydroxynonenal (4‐HNE) staining (a marker for ROS) in aortic plaques from ApoE^−/−^ mice, which was reduced with BH4 supplement (Figure 5F). These data suggest that nicotine induced endothelial dysfunction and accelerated the development of atherosclerosis and that BH4 supplement could reverse the endothelial dysfunction and attenuate atherosclerosis.

### 3.6 GTPCH1 overexpression reduces nicotine‐accelerated atherosclerosis

**Figure 6:**
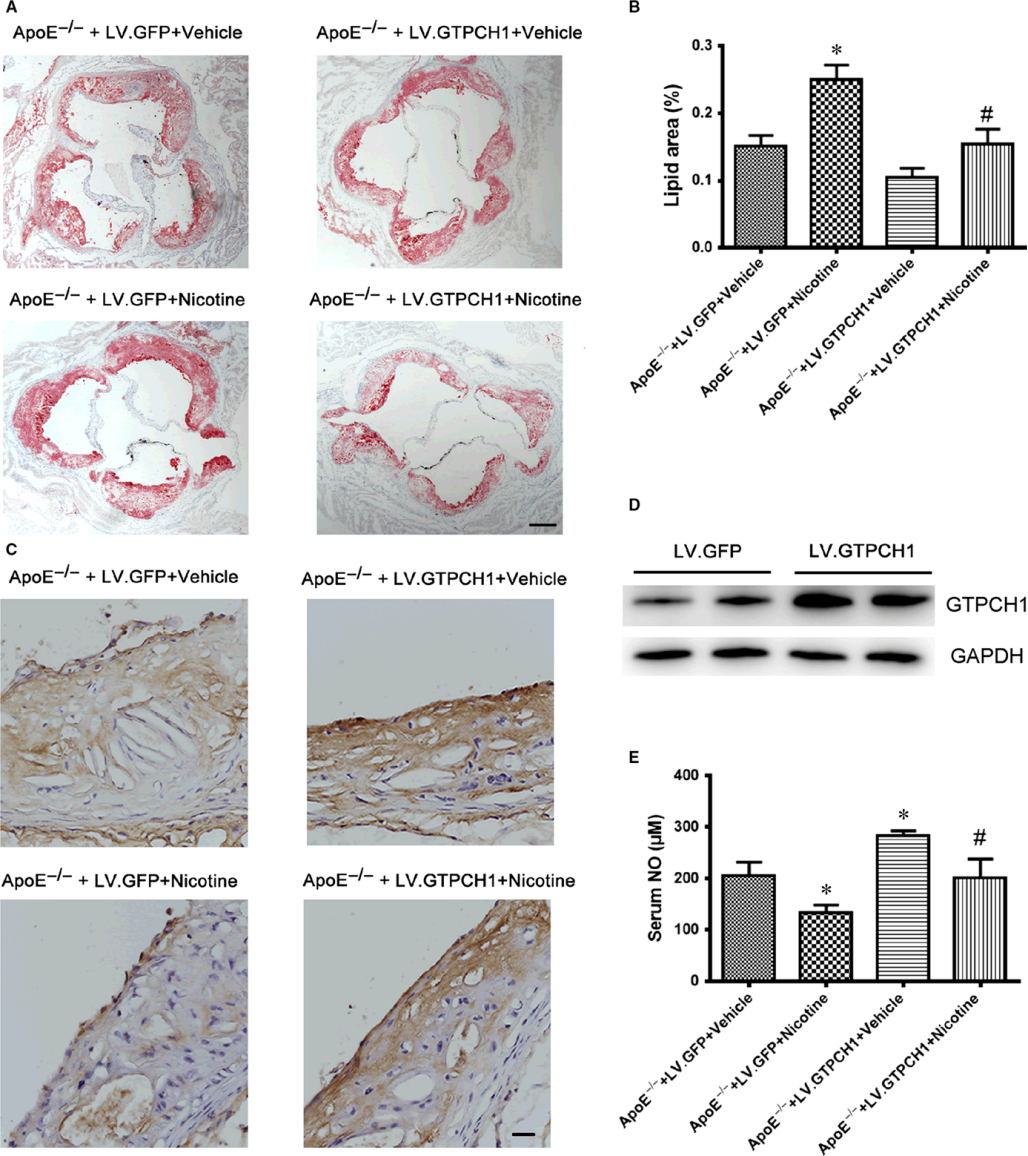
GTPCH1 overexpression reduces nicotine‐accelerated atherosclerosis. A, Oil‐red O staining of cross‐sectional aortic root lesions in 4 groups of mice. Scale bar = 100 μm. B, Quantitative analysis of aortic lesions expressed as percentage lesion area relative to total aorta area. n = 8 per group. *P < 0.05 vs ApoE^−/−^+LV.GFP+Vehicle. ^#^ *P* < 0.05 vs ApoE^−/−^+LV.GFP+Nicotine. C, Representative immunohistochemical staining of GTPCH1 in the aorta. Scale bar = 20 μm. D, Western blot analysis of aortic GTPCH1 level from mice injected with LV.GFP or LV.GTPCH1. E, Serum NO levels in the 4 groups of mice (n = 6). *P < 0.05 vs ApoE^−/−^+LV.GFP+Vehicle. ^#^ *P* < 0.05 vs ApoE^−/−^+LV.GFP+Nicotine. Data are mean ± SD

To evaluate the role of GTPCH1 in the development of atherosclerosis, we used lentivirus overexpression of GPF or GTPCH1 with LV.GFP or LV.GTPCH1 in ApoE^−/−^ mice fed an HFD with or without nicotine. The lesion size in aortic roots was greater in ApoE^−/−^ mice with than without nicotine and was reduced with GTPCH1 overexpression (Figure 6A and B). On immunohistochemistry, the expression of GTPCH1 in the aortic endothelium was reduced with nicotine treatment, and GTPCH1 overexpression in ApoE^−/−^ mice increased endothelial GTPCH1 level (Figure 6C). The protein expression of GTPCH1 in whole aorta was increased with LV.GTPCH1 injection compared with LV.GFP injection (Figure 6D), which further confirmed the efficiency of lentiviral transduction of GTPCH1 in vivo. Similarly, nicotine reduced serum NO level in mice, which was reversed with GTPCH1 overexpression (Figure 6E).

**Figure 7:**
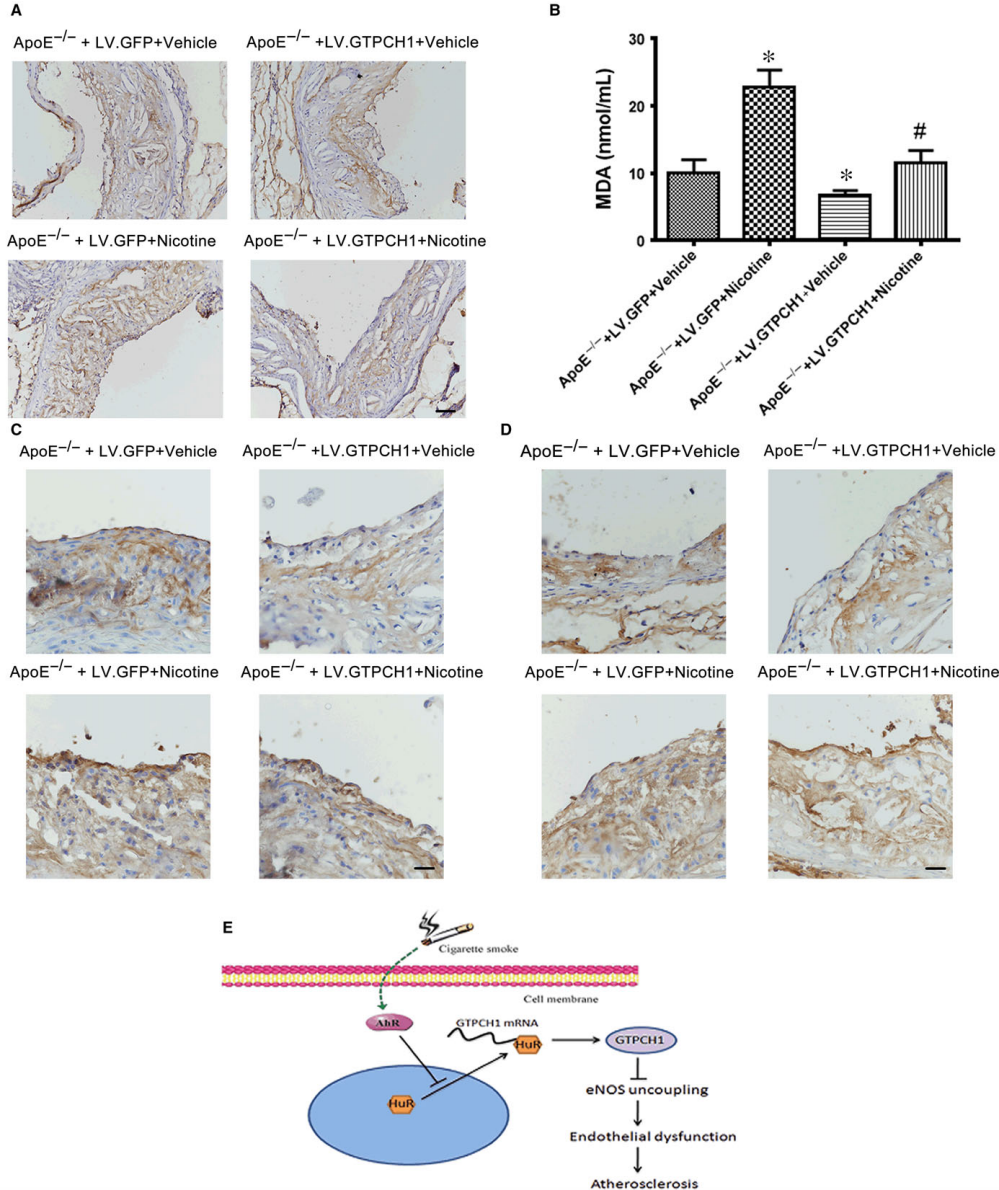
GTPCH1 overexpression reduces nicotine‐induced oxidant stress. A, Representative immunohistochemical staining of 4‐HNE in aortic plaques. Scale bar = 50 μm. B, malondialdehyde (MDA) level in the 4 groups of mice (n = 6). *P < 0.05 vs ApoE^−/−^+LV.GFP+Vehicle. ^#^ *P* < 0.05 vs ApoE^−/−^+LV.GFP+Nicotine. C and D, Representative immunohistochemical staining of ICAM1 and VCAM1 in aortic plaques. Scale bar = 20 μm. E, Diagram of nicotine‐induced endothelial dysfunction and atherosclerosis via HuR‐GTPCH1

Nicotine increased 4‐HNE staining, indicating ROS levels, in aortic plaques from ApoE^−/−^ mice, which was reduced with GTPCH1 overexpression (Figure 7A). Also, serum MDA level was increased with nicotine treatment and suppressed with GTPCH1 overexpression (Figure 7B). In the development of atherosclerosis, adhesion proteins induce the recruitment of monocytes,^16^ so we evaluated the effect of nicotine and GTPCH1 on the expression of adhesion proteins such as intracellular adhesion molecule 1 (ICAM1) and vascular cell adhesion molecule 1 (VCAM1). The levels of ICAM1 and VCAM1 were reduced with nicotine treatment and increased with GTPCH1 overexpression (Figure 7C and D). Taken together, nicotine induced eNOS uncoupling and accelerated the development of atherosclerosis, which was reduced by GTPCH1 overexpression.

## 4 DISCUSSION

In this study, we demonstrated that nicotine can induce endothelial dysfunction and promote the development of atherosclerosis. For the molecular mechanism, nicotine inhibits GTPCH1 expression by suppressing HuR translocation, thereby resulting in BH4 deficiency and eNOS uncoupling. BH4 supplement or GTPCH1 overexpression could reverse the endothelial dysfunction and reduce nicotine‐accelerated atherosclerosis. Our major finding is that GTPCH1 mRNA is a target of HuR. To our knowledge, this is the first evidence that nicotine mediates endothelial dysfunction by suppressing GTPCH1 expression.

GTPCH1 gene expression is regulated at transcription and post‐translation levels by the ubiquitin‐proteasome pathway and phosphorylation.^17^, ^18^, ^19^ Recently, GTPCH1 was also reported to be regulated by microRNA 133a.^20^ In this study, we demonstrated that HuR could bind with GTPCH1 mRNA and increase its mRNA stability, which is the first evidence that GTPCH1 mRNA stability is regulated by HuR. This discovery uncovered a novel mechanism of GTPCH1 gene regulation and also broadens the biological functions of HuR. Although HuR endothelium‐specific knockout mice were reported to regulate postnatal angiogenesis,^21^ the authors did not analyse the eNOS activity after HuR deletion in endothelial cells. We will detect it in our future study.

Accumulating data indicate that oxidant stress plays an important role in the beginning of cardiovascular diseases. In endothelial cells, oxidant stress from eNOS uncoupling mediates endothelial dysfunction in mice with diabetes, atherosclerosis and angiotensin II‐induced aneurysm.^22^, ^23^, ^24^ Thus, how oxidative stress is produced as an early and common pathogenic phenomenon in cardiovascular risk factors remains to be established. In this study, we found that GTPCH1 inhibition resulted in eNOS uncoupling and oxidant stress, which contributed to endothelial dysfunction and atherosclerosis. This finding was consistent with the phenotype of GTPCH1‐knockout mice.^7^ Therefore, GTPCH1 downregulation in endothelial cells might be a common mechanism for endothelial dysfunction during the development of cardiovascular diseases. Except in endothelial cells, GTPCH1 in vascular smooth muscle cells or leucocytes also participates in the development of atherosclerosis. Oxidized LDL inhibited GTPCH1 gene expression in interleukin‐1 beta activated vascular smooth muscle cells, which was postulated that diminished availability of BH4 may additionally impair the generation of NO in atherosclerosis.^25^ Macrophage‐specific GTPCH1 deficiency resulted in increased foam cell formation and altered cellular redox signalling, with decreased expression of antioxidant genes and increased reactive oxygen species in the atherosclerotic mice model.^26^ Thus, GTPCH1 plays an important protective role in the cardiovascular diseases such as atherosclerosis.

Smoking is a major preventable risk factor for the cardiovascular and metabolic diseases. Our previous studies demonstrated that nicotine, the major chemical of cigarette smoke, induced insulin resistance and the formation of abdominal aortic aneurysm in mice in vivo.^27^, ^28^ Also, cigarette smoke was reported to contribute to atherosclerosis, in which some mechanisms were revealed.^29^ For instance, nicotine was related to induced thrombosis, dyslipidemia and vascular inflammation.^30^, ^31^ In this study, we found that nicotine suppressed HuR translocation, which resulted in reduced GTPCH1 level and endothelial dysfunction. More importantly, BH4 supplement or GTPCH1 overexpression could improve endothelial function and reduce nicotine‐accelerated atherosclerosis. Our discovery uncovers a novel mechanism of nicotine in the development of atherosclerosis. In addition to atherosclerosis, the nicotine‐GTPCH1 signalling pathway may participate in other diseases such as abdominal aortic aneurysm and diabetes.

In summary, this study uncovered a novel mechanism by which nicotine induces endothelial dysfunction and accelerates atherosclerosis. BH4 supplement or GTPCH1 overexpression could reverse the endothelial dysfunction and reduce nicotine‐promoted atherosclerosis. Thus, targeting GTPCH1 should be an attractive strategy to clinically improve endothelial function in patients with cardiovascular diseases.

## CONFLICT OF INTEREST

The authors confirm that there are no conflicts of interest.

## AUTHOR CONTRIBUTION

J.L., S.L., G.C. and Y.S. designed and performed the research, J.L., W.C., F.D. and J.X. analysed data, C.Z. and W.Z. conceived the project, reviewed the data, and wrote the manuscript.
