# Supplementary material for: Nicotine induces endothelial dysfunction and promotes atherosclerosis via GTPCH1
Source: J Cell Mol Med. 2018 Aug 9;22(11):5406–17. doi: 10.1111/jcmm.13812 (PMC6201367; doi:10.1111/jcmm.13812)
Supplement: Supplementary file 1 [file JCMM-22-5406-s001.docx]

**Supplement**


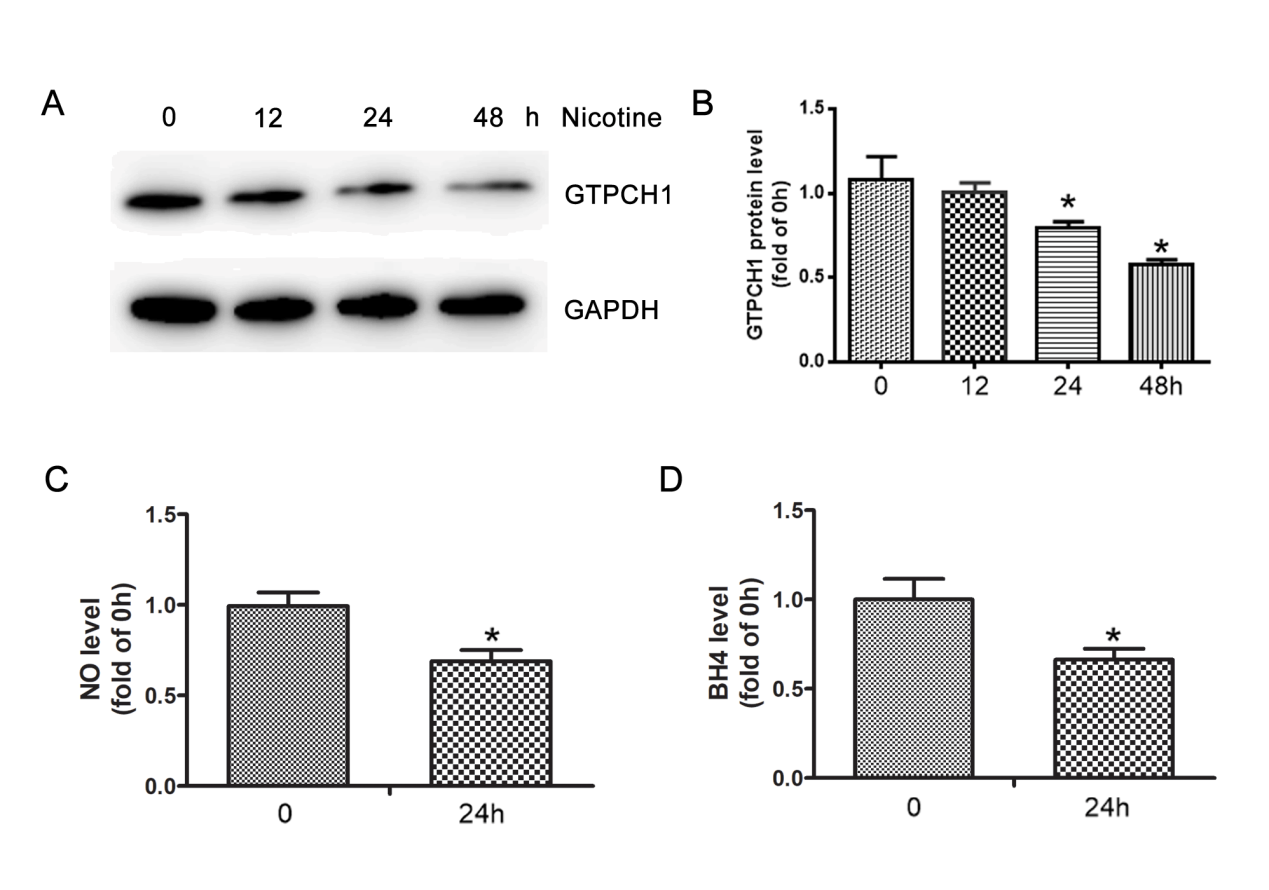


**Supplementary Figure 1. Nicotine inhibits GTPCH1 expression in endothelial cells with low oxygen.** **A,** Western blot analysis of GTPCH1 expression in HUVECs cultured in the 5% O_2_ incubator treated with 1 μM nicotine for different times and **B**, quantitative analysis (n=4). *p<0.05 *vs* 0 hr. **C**, NO level in HUVECs cultured in the 5% O_2_ incubator treated with nicotine for 24 hr (n=3). *p<0.05 vs 0 hr. **D**, BH4 level in HUVECs cultured in the 5% O_2_ incubator treated with nicotine for 24 hr (n=3). *p<0.05 vs 0 hr.
